# Cancer incidence in Kenya 1957-63.

**DOI:** 10.1038/bjc.1967.54

**Published:** 1967-09

**Authors:** C. A. Linsell


BRITISH JOURNAL OF CANCER

VOL. XXI SEPTEMBER, 1967 NO. 3

### CANCER INCIDENCE IN KENYA 1957–63

C. A. LINSELL

*From the Medical Research Laboratory, Nairobi, Kenya*

Received for publication November 30, 1966

HOFFMAN in 1915 stated confidently that " when every reasonable allowance is made for the want of accuracy and completeness in the available returns for the African Continent, it would seem safe to assume that cancer is of a relatively very low degree of frequency in African countries. even among the white population of European origin and that among the native population, as a general rule, malignant disease is extremely rare ". A steady flow of information from Africa, particularly in the last two decades, has shown that cancer in Africa is far from rare, although very different from the pattern seen in Europe and North America. A study of the cancer pattern of Southern Uganda, using the records of the Kampala hospital from 1897–1956 by Davies *et al.* (1964), indicated that the type and site pattern of cancer had not changed substantially over the years. The enthusiasm to record cancer patterns in Africa has been stimulated by the discovery of unusual situations, the investigation of which may add considerably to our knowledge of neoplasia. Early surveys were relative frequency studies of hospital or laboratory biopsy material which enabled comparison with other African or developing countries to be made. These studies contained many biases of collection and comparison with surveys from countries with more sophisticated vital statistics was excluded. Many objections to relative frequency ratio studies in Africa have been made; the reluctance of the public to use available medical facilities, illustrated by the preponderance of male patients in African hospitals, the varying medical facilities which are not themselves related absolutely to population density, the influence that the enthusiasm of medical staff in under-doctored areas can play in the detection of a specific disease and, above all, the natural preponderance of cancers readily available to the surgical knife in a poorly equipped peripheral hospital.

However, none of these biases explained the impressive percentage of some tumours in early reports from Africa—primary cancer of the liver in Portuguese East Africa, Prates (1943), the African lymphoma, Burkitt (1958) and naso-pharyngeal cancer in Kenya, Clifford (1961). There are now reliable rate surveys from Southern Africa, Nigeria, Portuguese East Africa and Uganda from which we can assess the over-all incidence of cancer and of specific sites against the background of developed countries. Edington and Maclean (1965) in a recent study in Ibadan comparing a relative ratio frequency survey with a rate study of the same population, concluded that if the source of bias is recognized the frequency survey can give a general pattern of malignancy in the community. That political frontiers in Africa are not limits of local pathology has been stressed by Hutt and Burkitt (1965) and they maintain that information for all sources must be accepted and evaluated to fill in the pattern of neoplasia in Africa.

It is therefore with this in mind that the experience of the Kenya Cancer Registry at the Medical Research Laboratory, Nairobi, is presented and the cancer pattern of Kenya and its neighbours discussed.

### MATERIAL AND METHODS OF THE STUDY

A registry of all cancer material was set up in 1961 at the Medical Research Laboratory, Nairobi. This is the central government laboratory responsible for histological examinations for all government hospitals in Kenya. There is a private laboratory in Nairobi, but during the years of this study, the work there concerned, almost exclusively, Kenyans other than those of African origin.

The present study reports only on Kenyans of African origin, although the Medical Research Laboratory has patients of other ethnic groups, mainly civil servants. The study was partly retrospective 1957–60 and then prospective from 1961–63 inclusive.

A Cope Chatterson—key sort—card system was adopted to record the following information: sex, age, tribe and district where the patient was first treated, site and histologic type of neoplasm and clinical history. The clinical records of the cases available in the laboratory were augmented by study of hospital records and further reports were requested from clinicians in charge of prospective cases. Retrospective cases which could not fulfil the simple criteria of the card system were eliminated. Biopsy registers and hospital records were checked against nominal rolls of the Cancer Registry to ensure that double registration did not occur following repeat biopsy. Histologic diagnosis had been made over the years of the survey by a number of pathologists, so when the clinical records had been completed the histology of all cases admitted to the registry was reviewed by the author.

Kenyans of African origin are members of four main ethnic groups: Bantu, Nilo-Hamitic, Nilotic and Hamitic. These ethnic groups are disproportionally represented in the population and tribal grouping is more clearly defined. The tribal groups represent approximate geographical areas and even when living away from this traditional home, tribal customs are preserved, so it was thought valuable to accept this parameter of local variation of cancer frequencies. Tribal surnames are distinctive so that even where the patient's tribe was not recorded, a reliable assumption could be made. A committee of laboratory workers from different tribal groups was formed to advise, when necessary, on this point.

To enable the tumour frequencies of the various tribal groups to be compared, a rate for the seven year period was calculated by division of the total number of tumours for individual sites by a standard population of seven tribal groups compiled from the 1963 census.

### RESULTS OF THE SURVEY

The trend of the total number of cases admitted over the seven year period showed a twofold increase by the end of the study, with no change in the sex ratio. The rise was most marked in the later years of the study when the cases were considered prospectively. This may have been due, in part, to an increased number of those tumours which were under active investigation by local clinicians. Nasopharyngeal and oesophageal tumours increased sixfold. However, lymphomas, which had received equal, if not more consideration, kept in line with the general trend only doubling in number. With the exception of the nasopharyngeal and oesophageal tumours, there were no major differences between the early retrospective study, and the later prospective material, and the time trend is related to an increased interest by local clinicians in cancer cases generally, and the establishment of a wider biopsy service and the cancer registry.

A total of 4206 patients were admitted to the study; 2305 males and 1901 females. The accurate assessment of age of African patients is difficult and, as population data was not available and the material was considered incomplete, no age-specific rates were attempted. However, considerable effort was made even with retrospective cases to ensure that tumours of children, defined as 15 years or under, could be differentiated. Among the patients admitted, there were 486 children—303 male and 183 female.

Following histologic diagnosis, the cases were classified by age, adult or child, and sex, using the WHO International Classification of Diseases, 1955 (Table I).

*Distribution of tumours by tribe*

The analysis, using the rate defined above, of the tumours by tribe, is shown in Table II. The gross rate by tribal group varied from 42/100,000 over the seven year period for the Kalenjin to 76/100,000 for the Coastal Bantu tribes.

*Relative frequencies in East Africa*

A comparison of relative frequencies of tumours classified by the International Classification of Diseases from Tanganyika (now part of Tanzania), Uganda and Kenya, was made using approximately the same number of tumours collected over the same period. The frequencies from Tanganyika were constructed from the Annual Reports of the Central Laboratory, Dar es Salaam, to fit the International Classification of Diseases used by Kenya in the present study and by Davies (1965) in Uganda. The period covered by the Uganda report is two years longer than that for Tanganyika and Kenya but the total number of tumours is approximately the same (Table III).

### DISCUSSION

*Cancer frequencies within Kenya*

The results have been presented in a form which enable comparison to be made with similar surveys and comment will be restricted to Table II showing sites or tumours with a significantly large number of cases to allow comparison between tribal groups.

The Coastal Bantu have the highest total tumour rate for the seven years (76/100,000) and Kisii and Kalenjin groups the lowest (43 and 42/100,000). This may indicate that case retrieval was not uniform.

The Coastal tribes have a cervical cancer rate 2–3 times higher than the other tribal groups. Circumcisional status and its relation to cancer of the penis and cervix have been considered by Dodge and Linsell (1963), and although a definite relationship was demonstrated between circumcision and cancer of the penis no correlation was seen between the two cancers. Circumcision is practised through-

TABLE I.—*Tumours Registered* 1957–63 *With WHO International Classification and Relative Frequencies*

| No. | Site | Male Adult | Male Child | % total male tumours | Female Adult | Female Child | % total female tumours | Total | % |
|---|---|---|---|---|---|---|---|---|---|
| 141 | Tongue | 12 | — | 0·5 | 14 | — | 0·7 | 26 | 0·6 |
| 142 | Salivary glands: | | | | | | | | |
| | Malignant | 18 | — | 0·8 | 16 | 1 | 0·9 | 35 | 0·8 |
| | Mixed | 42 | 3 | 2·0 | 61 | 3 | 3·4 | 109 | 2·6 |
| 143, 144 | Mouth | 10 | — | 0·4 | 19 | — | 1·0 | 29 | 0·7 |
| 145 | Tonsil | 3 | — | 0·1 | 1 | — | | 4 | <0·1 |
| 146 | Nasopharynx | 88 | 9 | 4·2 | 26 | 1 | 1·4 | 124 | 2·9 |
| 150 | Oesophagus | 175 | — | 7·6 | 10 | — | 0·5 | 185 | 4·4 |
| 151 | Stomach | 57 | — | 2·5 | 43 | — | 2·3 | 100 | 2·3 |
| 152 | Small intestine | 5 | 1 | 0·3 | 2 | — | | 8 | 0·2 |
| 153 | Large intestine | 42 | — | 1·8 | 27 | — | 1·4 | 69 | 1·6 |
| 154 | Rectum | 26 | 1 | 1·2 | 10 | — | 0·5 | 37 | 0·9 |
| 155 | Liver | 160 | — | 7·0 | 57 | 1 | 3·0 | 218 | 5·2 |
| | Gall bladder | 11 | — | 0·5 | 8 | — | 0·4 | 19 | 0·5 |
| 156 | Liver, secondary | 17 | 1 | 0·8 | 5 | 1 | 0·3 | 24 | 0·6 |
| 157 | Pancreas | 12 | — | 0·5 | 6 | — | 0·3 | 18 | 0·4 |
| 158 | Peritoneum | 1 | — | | 3 | — | 0·2 | 4 | <0·1 |
| 159 | Digestive organs, unspecified | 5 | — | 0·2 | 4 | — | 0·2 | 9 | 0·2 |
| 160 | Nasal cavity and sinuses | 31 | 2 | 1·4 | 20 | 1 | 1·1 | 54 | 1·3 |
| 161 | Larynx | 12 | — | 0·5 | 5 | 1 | 0·3 | 18 | 0·4 |
| 162 | Lung, bronchus and trachea | 24 | — | 1·0 | 7 | — | 0·4 | 31 | 0·7 |
| 163 | Lung, secondary | 4 | — | 0·2 | 1 | — | | 5 | 0·1 |
| 170 | Breast | 23 | — | 1·0 | 174 | — | 9·2 | 197 | 4·7 |
| 171 | Cervix uteri | | | | 270 | — | 14·2 | 270 | 6·4 |
| 172, 174 | Uterus | | | | 23 | — | 1·2 | 23 | 0·6 |
| 173 | Choriocarcinoma | | | | 47 | — | 2·5 | 47 | 1·1 |
| 175 | Ovary, tubes | | | | 49 | 2 | 2·7 | 51 | 1·2 |
| 176 | Vagina | | | | 9 | — | 0·4 | 9 | 0·2 |
| | Vulva | | | | 24 | 1 | 1·3 | 25 | 0·6 |
| | Unspecified | | | | 8 | — | 0·4 | 8 | 0·2 |
| 177 | Prostrate | 44 | — | 1·9 | | | | 44 | 1·0 |
| 178 | Testis | 10 | — | 0·4 | | | | 10 | 0·2 |
| 179 | Penis, scrotum | 42 | — | 1·8 | | | | 42 | 1·0 |
| 180 | Kidney: Wilms | 2 | 10 | 0·5 | — | 18 | 0·9 | 30 | 0·7 |
| | Other | 6 | 2 | 0·4 | 8 | — | 0·4 | 16 | 0·4 |
| 181 | Bladder, urethra | 29 | 1 | 1·3 | 13 | 1 | 0·7 | 44 | 1·0 |
| 190 | Melanoma, skin | 94 | — | 4·1 | 100 | — | 5·3 | 194 | 4·6 |
| 191 | Skin, Squamous | 267 | 4 | 11·8 | 300 | 5 | 16·0 | 576 | 13·7 |
| | Skin, basal | 8 | — | 0·3 | 14 | — | 0·7 | 22 | 0·6 |
| 192 | Eye | 30 | 24 | 2·3 | 21 | 15 | 1·9 | 90 | 2·1 |
| 193 | Brain | 12 | 3 | 0·6 | 4 | 5 | 0·5 | 24 | 0·6 |
| 194 | Thyroid | 21 | 1 | 0·9 | 21 | 1 | 1·2 | 44 | 1·0 |
| 195 | Other endocrine | 2 | 1 | 0·1 | — | — | | 3 | <0·1 |
| 196 | Bone, sarcoma | 40 | 5 | 2·0 | 27 | 6 | 1·7 | 78 | 1·8 |
| | Osteoclastoma | 8 | 3 | 0·5 | 8 | 5 | 0·7 | 24 | 0·6 |
| 197 | Connective tissue | 103 | 11 | 5·0 | 68 | 9 | 4·0 | 291 | 6·9 |
| | Kaposi | 92 | 4 | 4·2 | 14 | 1 | 0·8 | 111 | 2·6 |
| 198 | Glands, secondary | 126 | 4 | 5·6 | 57 | 2 | 3·1 | 189 | 4·5 |
| 199 | Unspecified site | 35 | 1 | 1·6 | 29 | 4 | 1·7 | 69 | 1·6 |
| 200·0 | Reticulosarcoma | 61 | 12 | 3·2 | 18 | 6 | 1·3 | 97 | 2·3 |
| 200·1 | Lymphosarcoma | 122 | 154 | 12·0 | 46 | 82 | 6·7 | 404 | 9·6 |
| 201 | Hodgkins disease | 44 | 41 | 3·7 | 14 | 9 | 1·2 | 108 | 2·6 |
| 202 | Reticulosis | 3 | 1 | 0·2 | 1 | — | | 5 | 0·1 |
| 203 | Myeloma | 15 | — | 0·6 | 3 | — | 0·2 | 18 | 0·4 |
| 204 | Leukaemia | 7 | 3 | 0·4 | 3 | 2 | 0·3 | 15 | 0·4 |
| 205 | Mycosis fungoides | 1 | 1 | | — | — | | 2 | <0·1 |
| | Totals | 2002 | 303 | 2305 | 1718 | 183 | 1901 | 4206 | |

TABLE II.—*Tumours Ranked by Tribe and Sex*

| | Abalulya | | | Luo | | | Kisii | | | Kalenjin | | | Kikuyu | | | Kamba | | | Coast | | |
|---|---|---|---|---|---|---|---|---|---|---|---|---|---|---|---|---|---|---|---|---|---|
| | M | F | T | M | F | T | M | F | T | M | F | T | M | F | T | M | F | T | M | F | T |
| 1. Skin squamous | 4·1 | 1·6 | 5·7 | 4·2 | 2·2 | 6·4 | 3·4 | 5·0 | 8·4 | 3·0 | 3·8 | 6·8 | 3·4 | 6·0 | 9·4 | 3·2 | 6·9 | 10·1 | 5·7 | 3·3 | 9·0 |
| 2. Lymphosarcoma | 3·9 | 2·1 | 6·0 | 4·7 | 2·9 | 7·6 | 2·0 | 2·4 | 4·4 | 1·8 | 0·4 | 2·2 | 3·1 | 1·3 | 4·4 | 4·6 | 2·1 | 6·7 | 7·5 | 2·5 | 10·0 |
| 3. Cervix | — | 2·4 | 2·4 | — | 3·5 | 3·5 | — | 2·4 | 2·4 | — | 2·0 | 2·0 | — | 4·5 | 4·5 | — | 3·6 | 3·6 | — | 7·2 | 7·2 |
| 4. Liver | 1·4 | 0·4 | 1·8 | 2·0 | 0·8 | 2·8 | 0·8 | — | 0·8 | 1·1 | 0·6 | 1·7 | 2·0 | 1·2 | 3·2 | 5·8 | 1·1 | 6·9 | 2·2 | 1·2 | 3·4 |
| 5. Breast | 0·3 | 1·5 | 1·8 | — | 1·8 | 1·8 | 0·4 | 1·0 | 1·4 | 0·2 | 2·0 | 2·2 | 0·4 | 3·7 | 4·1 | 0·5 | 1·7 | 2·2 | 0·2 | 2·2 | 2·4 |
| 6. Connective tissue | 1·3 | 0·5 | 1·8 | 1·4 | 0·9 | 2·3 | 2·4 | 1·2 | 3·6 | 1·4 | 1·1 | 2·5 | 1·5 | 1·0 | 2·5 | 1·8 | 1·4 | 3·2 | 1·8 | 2·3 | 4·1 |
| 7. Melanoma | 1·4 | 1·1 | 2·5 | 1·0 | 0·4 | 1·4 | 1·0 | 1·8 | 2·8 | 1·0 | 1·5 | 2·5 | 1·2 | 1·5 | 2·7 | 2·5 | 2·0 | 4·5 | 2·2 | 1·2 | 3·4 |
| 8. Glands secondary | 2·3 | 0·8 | 3·1 | 1·3 | 0·4 | 1·7 | 1·8 | 0·8 | 2·6 | 2·9 | 1·3 | 4·2 | 1·7 | 1·0 | 2·7 | 0·7 | 0·7 | 1·4 | 2·0 | 0·8 | 2·8 |
| 9. Oesophagus | 2·7 | — | 2·7 | 6·0 | 0·4 | 6·4 | 1·0 | — | 1·0 | 0·4 | — | 0·4 | 2·7 | 0·2 | 2·9 | 0·6 | 0·1 | 0·7 | 1·2 | — | 1·2 |
| 10. Nasopharynx | 1·5 | — | 1·5 | 0·6 | 0·2 | 0·8 | 0·6 | 0·4 | 1·0 | 2·5 | 0·7 | 3·2 | 1·6 | 0·6 | 2·2 | 1·1 | 0·4 | 1·5 | 0·2 | — | 0·2 |
| 11. Kaposi | 1·8 | 0·2 | 2·0 | 1·4 | 0·2 | 1·6 | 0·8 | 0·2 | 1·0 | 0·8 | 0·2 | 1·0 | 1·6 | 0·4 | 2·0 | 1·3 | 0·1 | 1·4 | 1·2 | 0·2 | 1·4 |
| 12. Salivary—mixed | 0·3 | 1·0 | 1·3 | 0·8 | 0·5 | 1·3 | 0·2 | 1·0 | 1·2 | 1·0 | 0·5 | 1·5 | 0·8 | 0·9 | 1·7 | 0·3 | 1·3 | 1·6 | 0·8 | 1·2 | 2·0 |
| 13. Hodgkin's disease | 1·5 | 0·5 | 2·0 | 1·5 | 0·2 | 1·7 | 0·8 | 0·6 | 1·4 | 0·8 | — | 0·8 | 1·0 | 0·3 | 1·3 | 0·9 | 0·6 | 1·5 | 2·2 | 0·4 | 2·6 |
| 14. Stomach | 1·3 | 0·1 | 1·4 | 0·8 | 0·2 | 1·0 | 0·4 | 1·0 | 1·4 | 0·4 | 0·2 | 0·6 | 1·1 | 1·2 | 2·3 | 0·5 | 0·2 | 0·7 | 0·6 | 0·6 | 1·2 |
| 15. Bone | 0·5 | 0·6 | 1·1 | 0·6 | 0·4 | 1·0 | 0·6 | — | 0·6 | 1·1 | 0·6 | 1·7 | 0·8 | 0·8 | 1·6 | 1·0 | 1·0 | 2·0 | 1·2 | 0·4 | 1·6 |
| 16. Reticulosarcoma | 0·9 | 0·3 | 1·2 | 1·2 | 0·4 | 1·6 | 1·0 | 0·4 | 1·4 | 0·8 | — | 0·8 | 0·9 | 0·4 | 1·3 | 1·4 | 0·1 | 1·5 | 0·8 | 0·4 | 1·2 |
| Total tumours per 100,000 over 7 years | | 50 | | | 58 | | | 43 | | | 42 | | | 64 | | | 62 | | | 76 | |

M = Male F = Female T = Total

TABLE III.—*Comparison of Relative Frequencies of Cancer by Site for Tanganyika, Kenya and Uganda*

| I.C.D. No. | Tumour site | Tanganyika | Kenya | Uganda |
|---|---|---|---|---|
| | Period of study: | 1957–61 | 1957–61 | 1954–61 |
| | Total tumours: | 2940 | 2747 | 2926 |
| 140 | Lip | 0·0 | 0·0 | 0·3 |
| 141 | Tongue | 0·5 | 0·7 | 0·3 |
| 142 | Salivary gland—malign | 1·4 | 0·6 | 1·7 |
| | —benign | 3·5 | 2·5 | — |
| 143, 144 | Mouth | 2·1 | 0·4 | 0·5 |
| 145 | Tonsil | <0·1 | 0·1 | 0·1 |
| 146 | Nasopharynx | 0·0 | 2·3 | 0·1 |
| 148 | Pharynx | <0·1 | <0·1 | 0·1 |
| 150 | Oesophagus | 0·3 | 3·9 | 2·0 |
| 151 | Stomach | 0·9 | 2·5 | 2·9 |
| 152, 153 | Intestines | 0·7 | 2·4 | 1·4 |
| 154 | Rectum | 1·2 | 1·3 | 1·7 |
| 155 | Liver and biliary passages | 3·0 | 5·2 | 6·8 |
| 156 | Liver secondary | 0·2 | 0·5 | 1·4 |
| 157 | Pancreas | 0·1 | 0·5 | 0·6 |
| 158 | Peritoneum | 0·2 | 0·0 | 0·0 |
| 159 | Unspecified digestive organs | 0·0 | 0·1 | 0·0 |
| 160 | Nasal cavity and sinuses | 0·4 | 1·2 | 1·7 |
| 161 | Larynx | 0·2 | 0·4 | 0·4 |
| 162 | Lung | 0·0 | 0·8 | 0·8 |
| 163 | Lung secondary | <0·1 | 0·1 | 0·4 |
| 170 | Breast | 3·4 | 4·6 | 4·0 |
| 171 | Cervix uteri | 8·2 | 6·6 | 9·9 |
| 172 | Corpus uteri | 0·7 | 0·6 | 0·8 |
| 173, 174 | Uterus other and choriocarcinoma | 1·0 | 1·2 | 0·6 |
| 175 | Ovary, Fallopian tube | 2·1 | 1·3 | 3·5 |
| 176 | Vulva, vagina | 1·4 | 1·0 | 1·4 |
| 177 | Prostate | 1·9 | 1·0 | 2·5 |
| 178 | Testis | 0·3 | 0·2 | 0·1 |
| 179 | Penis, scrotum | 3·2 | 1·1 | 7·3 |
| 180 | Kidney | 0·8 | 1·0 | 1·0 |
| 181 | Bladder | 2·6 | 1·0 | 4·1 |
| 190 | Melanoma | 5·0 | 4·8 | 1·6 |
| 191 | Squamous | 16·3 | 15·2 | 4·9 |
| | Basal cell | 1·4 | 0·8 | — |
| 192 | Eye | 1·7 | 2·2 | 3·0 |
| 193 | Brain—C.N.S. | 0·2 | 0·5 | 1·1 |
| 194 | Thyroid | 1·3 | 1·0 | 1·3 |
| 195 | Other endocrine glands | 0·1 | <0·1 | 0·6 |
| 196 | Bone, Sarcoma | 1·1 | 0·8 | 1·9 |
| | Adamantinoma | 0·9 | 0·4 | 1·3 |
| | Osteoclastoma | 0·3 | 0·5 | — |
| 197 | Connective tissue sarcoma | 5·3 | 4·9 | 3·1 |
| | Kaposi's sarcoma | 4·7 | 2·4 | 4·1 |
| 198, 199 | Metastatic | 7·8 | 6·1 | 3·9 |
| 200 | Reticulum cell sarcoma | 4·6 | 2·5 | } 8·8 |
| | Lymphosarcoma | 5·2 | 9·6 | |
| 201 | Hodgkin's disease | 2·1 | 2·5 | 1·9 |
| 202 | Other lymphomas | 0·6 | 0·0 | 0·0 |
| 203 | Myeloma | 0·1 | 0·2 | 0·5 |

Sources: Tanganyika—Annual Reports of the Government Laboratories, Dar-es-Salaam, 1957–61.
Uganda—Davies, J. N. P., Knowelden, J. and Wilson, B. A. (1965) *J. natn. Cancer Inst.*, **35**, 5.
Kenya—Kenya Cancer Registry—present study.

out the Coastal region. The distribution of total lymphomas, excluding Hodgkins disease, conforms roughly to the limits for the Burkitt's tumour suggested by Uganda workers. Cases compatible with Burkitt's tumour have seen in Kenya in areas outside these limits and the sites of many of these cases were checked by Dalldorf, Linsell, Barnhart and Martyn (1964). Fig. 1 shows a comparison of adults and childhood lymphomas in Kenya. Although the exact definition of Burkitt tumour may evoke discussion, childhood lymphoma in Kenya has a definite tribal pattern. The pattern of Hodgkins disease however is much more uniform in the population. Hodgkins disease is unusually common in children in Kenya, 46 per cent of the cases presenting under 15 years of age. Azzam (1966) reviewing Hodgkins disease in Lebanese children recorded 22·5 per cent

FIG. —Lymphomas in Kenya excluding Hodgkin's disease. Scale—Total lymphomas/standard tribal population.

there as a high level and Solidoro, Guzmán and Chang (1966) from Peru reported 40·7 per cent. Although population structures differ widely from Europe and North America this cannot be the sole reason for such high levels.

The incidence of alimentary tract cancer has been reviewed by Linsell (1967) and the situation of oesophageal cancer among the Luo and Kalenjin tribes is indicated as deserving further epidemiological investigation. The contrast between the incidence of nasopharyngeal cancer of the Kalenjin and the Coastal Bantu has been discussed by Clifford and Beecher (1964).

*Cancer frequencies in East Africa*

Compared with Tanganyika and Uganda, salivary gland tumours in Kenya are less frequent. Salivary tumours in East Africa present very commonly in sites other than the parotid; in Kenya 46 per cent of these tumours occur in the accessory glands (Linsell and Clifford, 1964).

The high incidence of oral cancers in Tanganyika may result from the inclusion of Indian patients who were specifically excluded from the other two surveys. The nasopharynx appears to be exclusively of interest in Kenya and as nasal cavity cancers are equally represented it is unlikely that anatomical classification has any influence on the marked differences of nasopharyngeal cancer. The absence of gastro-intestinal cancer in Tanganyika requires further investigation to exclude obvious under-reporting as rectal carcinoma, more accessible to biopsy, is equal in the three countries.

The influence of circumcision and penile cancer in East Africa is very marked but it cannot be the sole factor as frequency within tribes who do not circumcise varies considerably. Dodge (1964) suggested that retention of urine following stricture was of more importance than bilharzia in the aetiology of bladder cancer in Uganda. Strictures are more common there than in Kenya but bladder cancer in Kenya is higher in those areas where bilharzia is a problem.

The low frequency of skin cancers both epithelioma and melanoma in Uganda also requires further investigation.

Epithelioma of the skin is the commonest cancer in Kenya and is usually considered to arise in scar tissue at the site of a healed ulcer or burn.

Cancer registration is continuing in East Africa and has been greatly stimulated by a current hospital survey by Burkitt (Hutt and Burkitt 1965).

This is a wide survey using mainly clinical material from hospitals both government and mission sponsored and the value of such surveys has been demonstrated by the number of interesting local situations which it has presented. More detailed and accurate registration can only be obtained by surveys similar to that of Davies in Kyadonda District of Uganda where the calculation of cancer rates requires a defined population with vital statistics and total collection of all cancer cases must be attempted. This is very expensive in Africa and creates its own problems, but it should be undertaken wherever possible. The exposure of interesting local situations is more likely if definite parameters are selected in prospective studies. Ethnic, tribal or political divisions such as districts and provinces can be used. Tribal differences are considered in Africa to be of outstanding value as intermarriage, isolation and unusual customs may play a revealing part in the genesis of their cancers. Hutt and Burkitt (1965) have stressed the necessity of a combined clinico-pathological approach to cancer problems in the field of East Africa. To this team it is suggested must be added the experience of epidemiology and sociology.

### REFERENCES

Azzam, S. A.—(1966) *Cancer Res.*, **26**, 1202.

Burkitt, D.—(1958) *Br. J. Surg.*, **46**, 218.

Clifford, P. (1961) *J. Lar. Otol.*, **53**, 558.

Clifford, P. and Beecher, J. L.—(1964) *Br. J. Cancer*, **18**, 25.

Dalldorf, G., Linsell, C. A., Barnhart, F. E. and Martyn, R.—(1964) *Perspect. Biol. Med.*, **7**, 435.

Davies, J. N. P., Elmes, S., Hutt, M. S. R., Intimaralye, L. A. R., Owor, R. and Shaper, L.—(1964) *Br. med. J.*, i, 259, 336.

Davies, J. N. P. Knowelden, J. and Wilson, B. A.—(1965) *J. natn. Cancer Inst.*, **35**, 5.

Dodge, O. G.—(1964) *Cancer, N.Y.*, **17**, 1433.

Edington, G. M. and Maclean, C. M. U.—(1965) *Br. J. Cancer*, **19**, 471.

HOFFMAN, F. L.—(1915) ' Mortality from Cancer throughout the World. ' New Jersey.
HUTT, M. S. R. AND BURKITT, D.—(1965) *Br. med. J.*, ii, 719.
LINSELL, C. A. AND CLIFFORD, P.—(1964) *E. Afr. med. J.*, **41**, 263.
LINSELL, C. A.—(1967) *J. natn. Cancer Inst.*, in press.
PRATES, M. D.—(1943) 'Contribuição para o Estudo da Etiologia e Patogenia dos Tumores Malignos do Fígado dos Indígenas de Moçambique'. Thesis, University of Lisbon.
SOLIDORO, A., GUZMÁN, C. AND CHANG, A.—(1966) *Cancer Res.*, **26**, 1204.


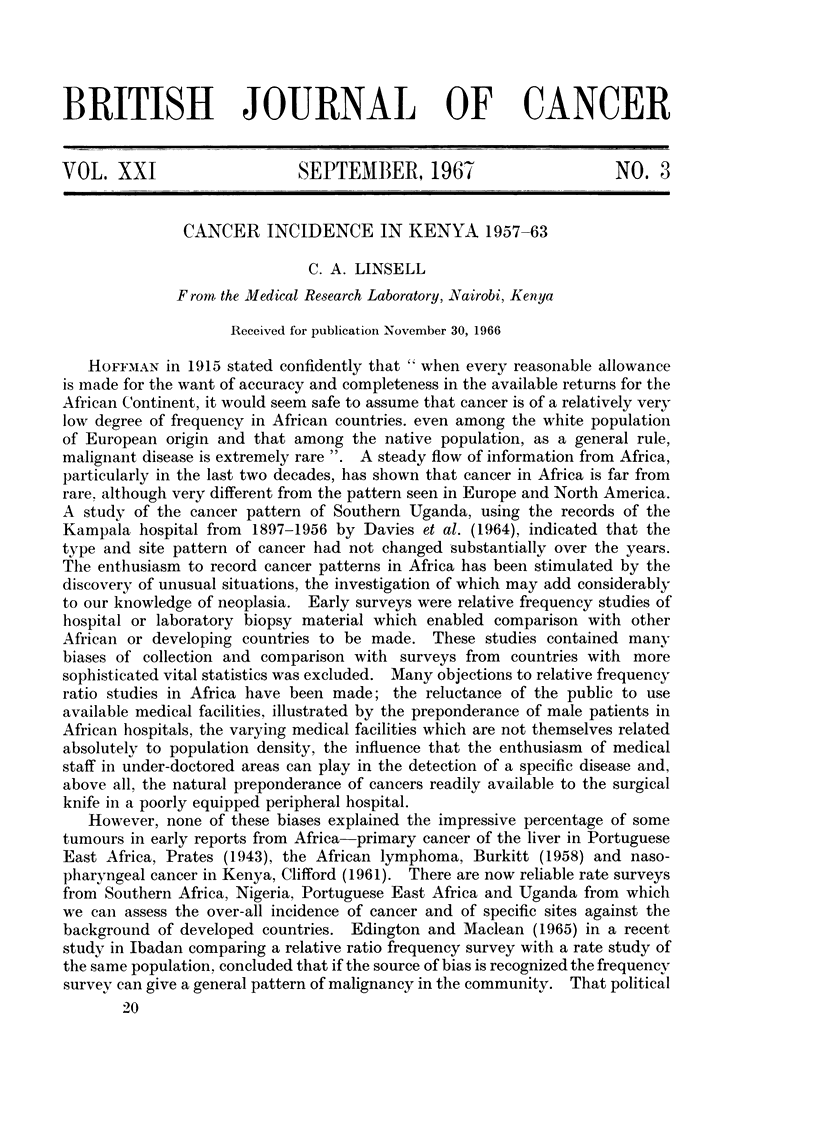

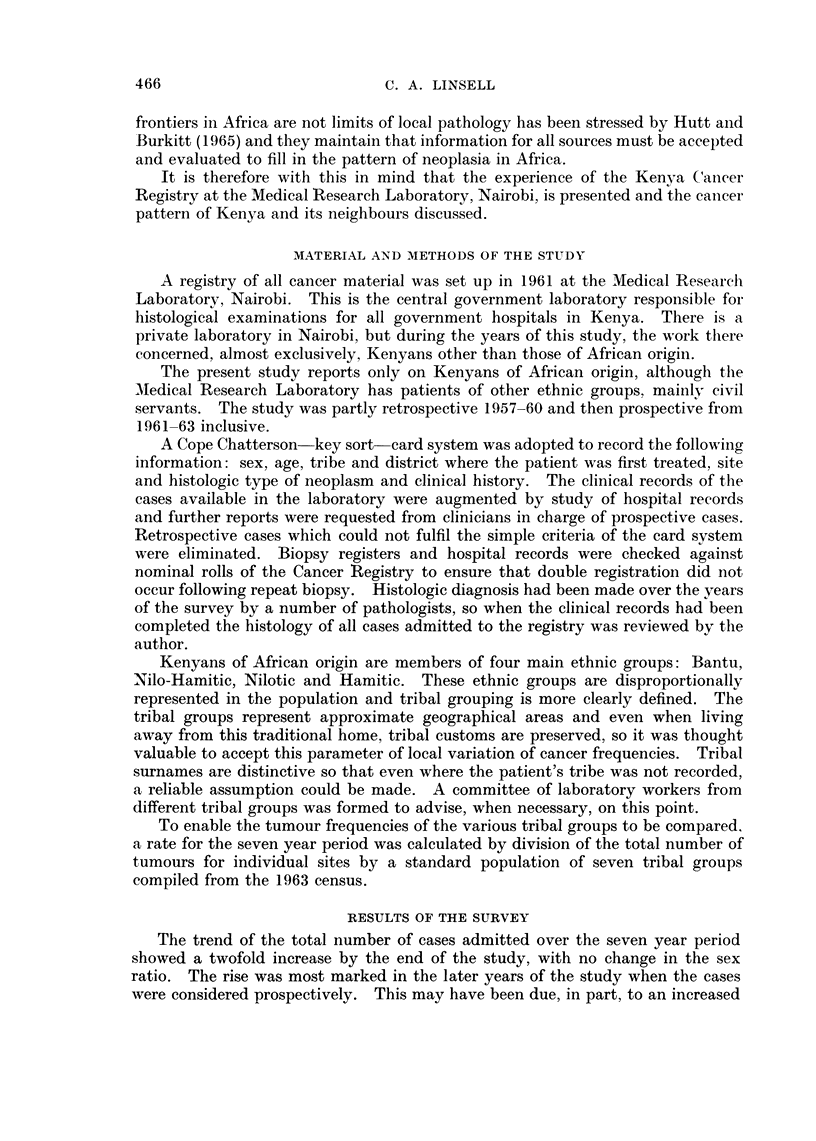

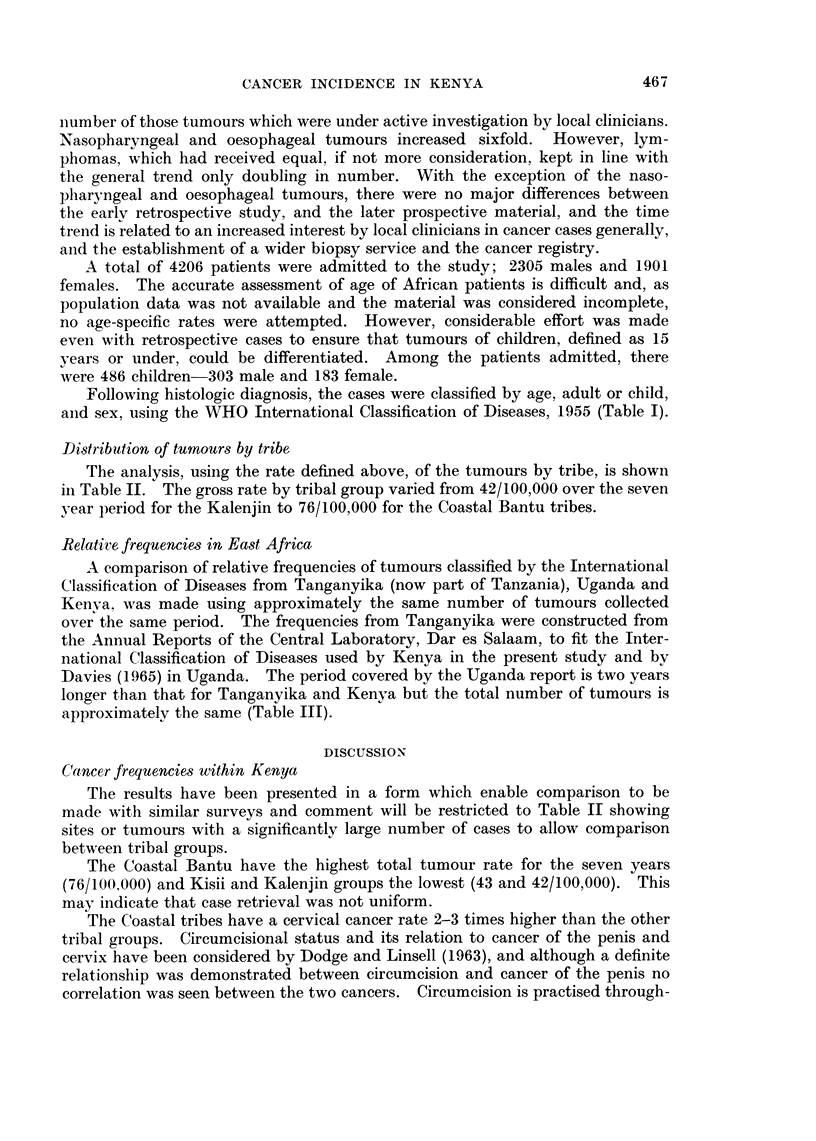

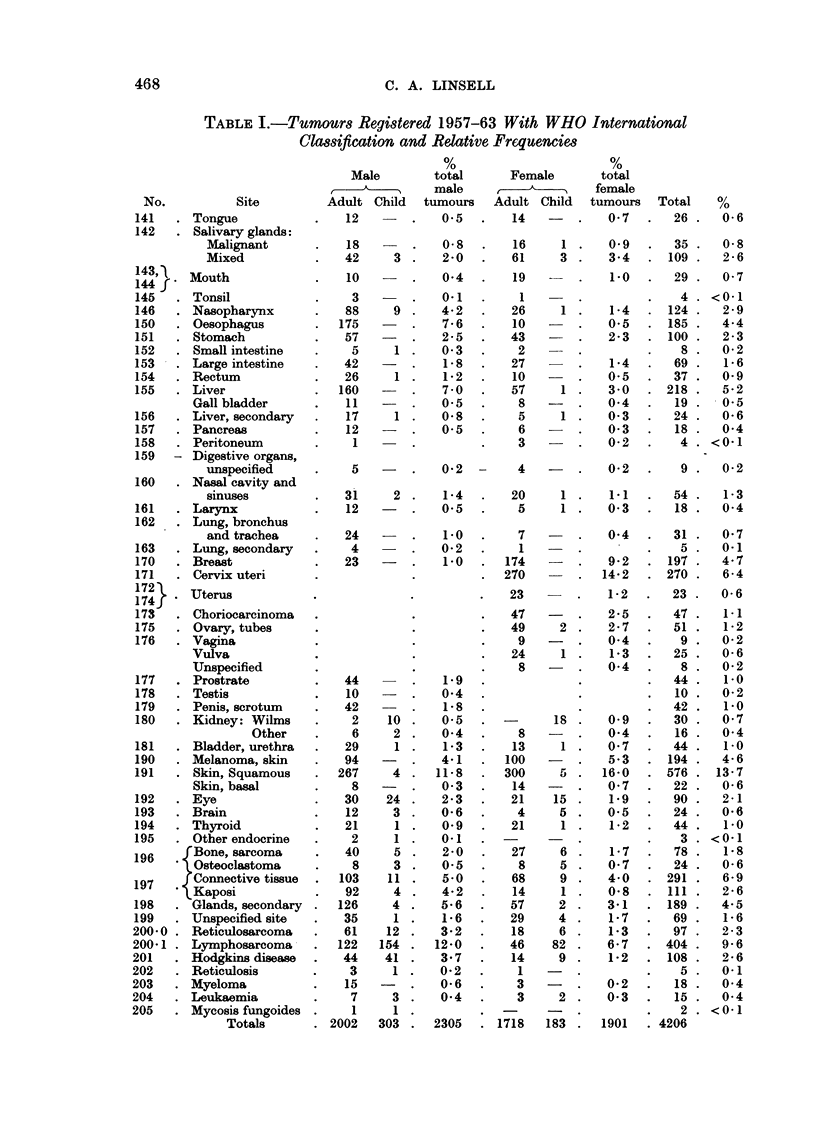

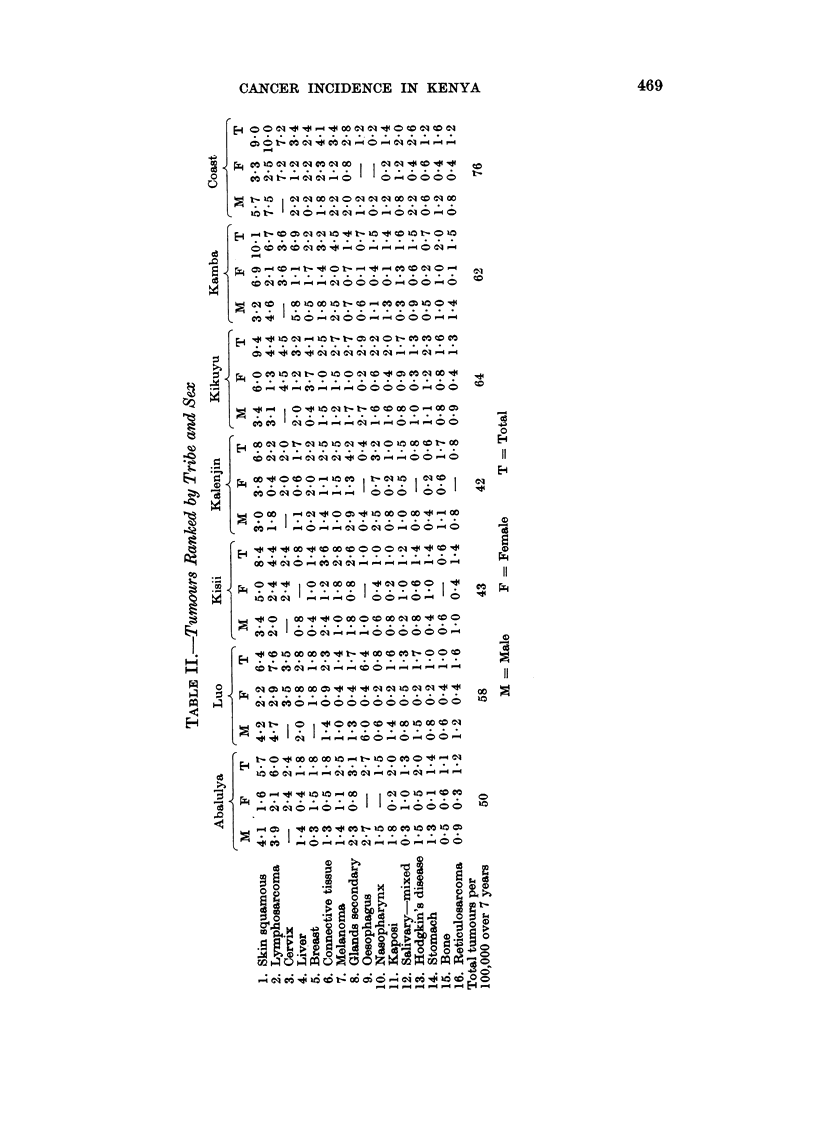

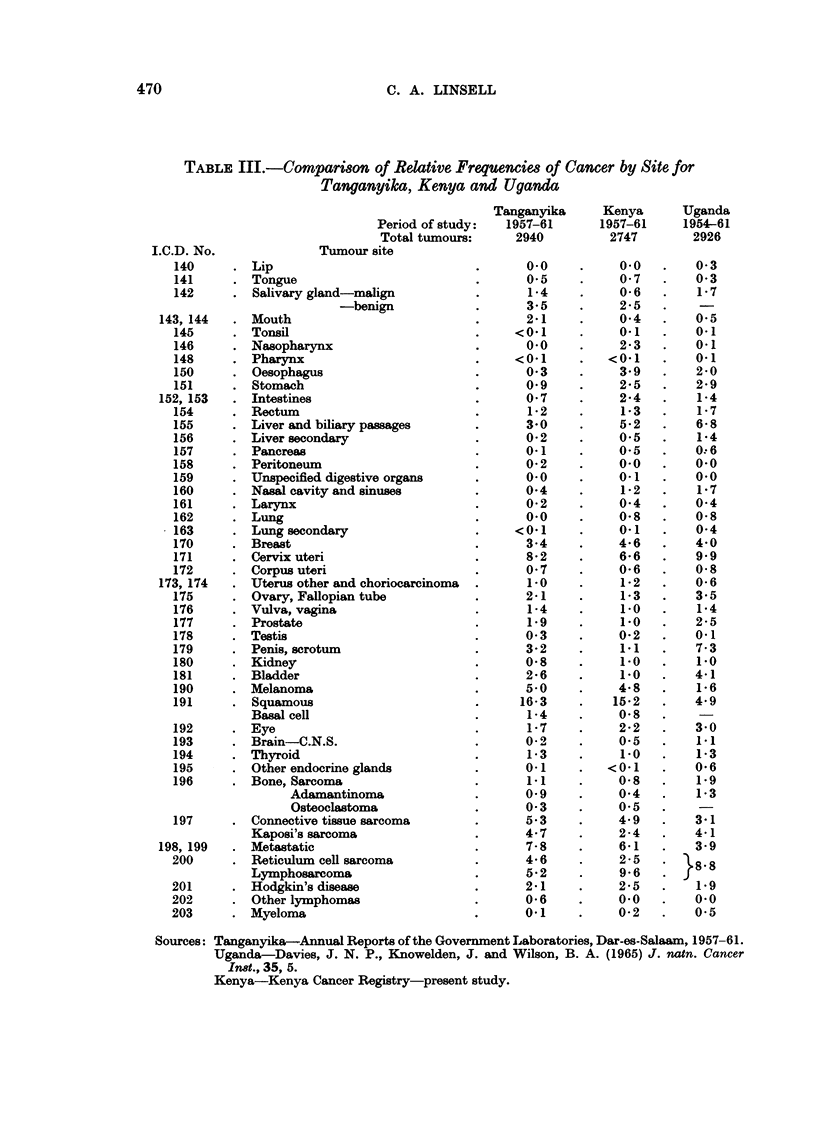

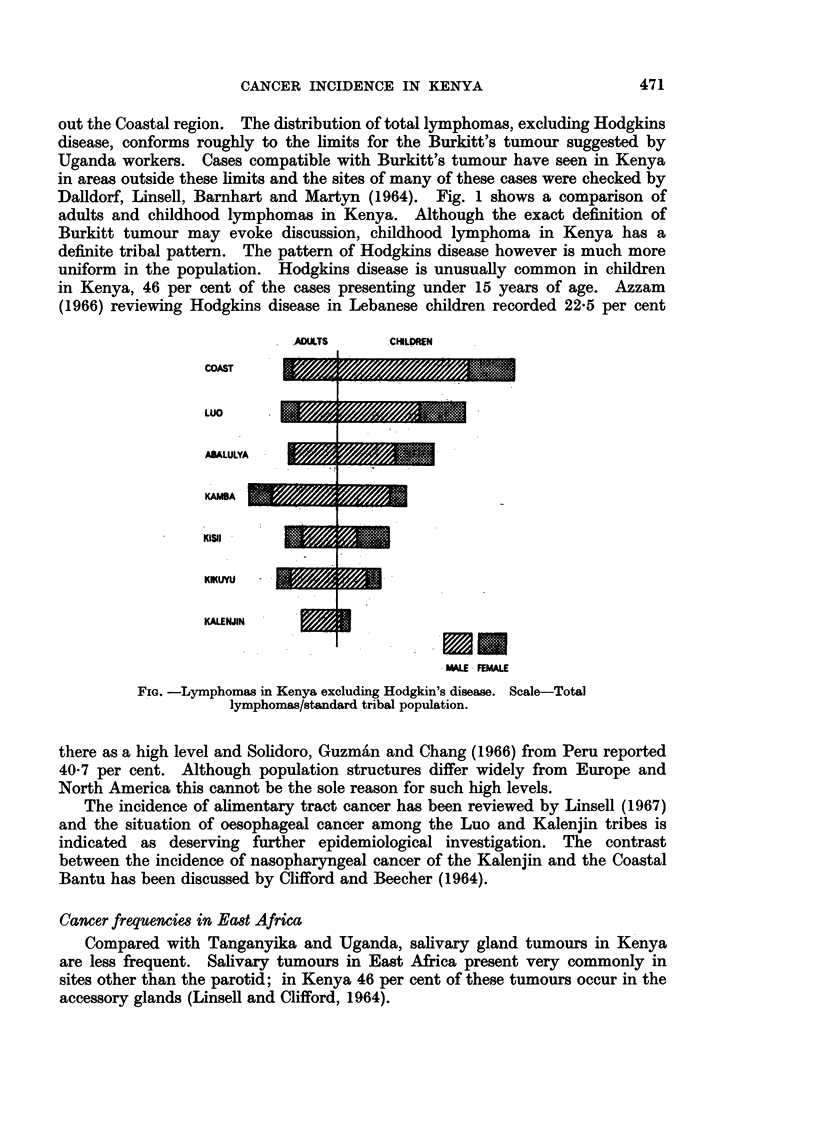

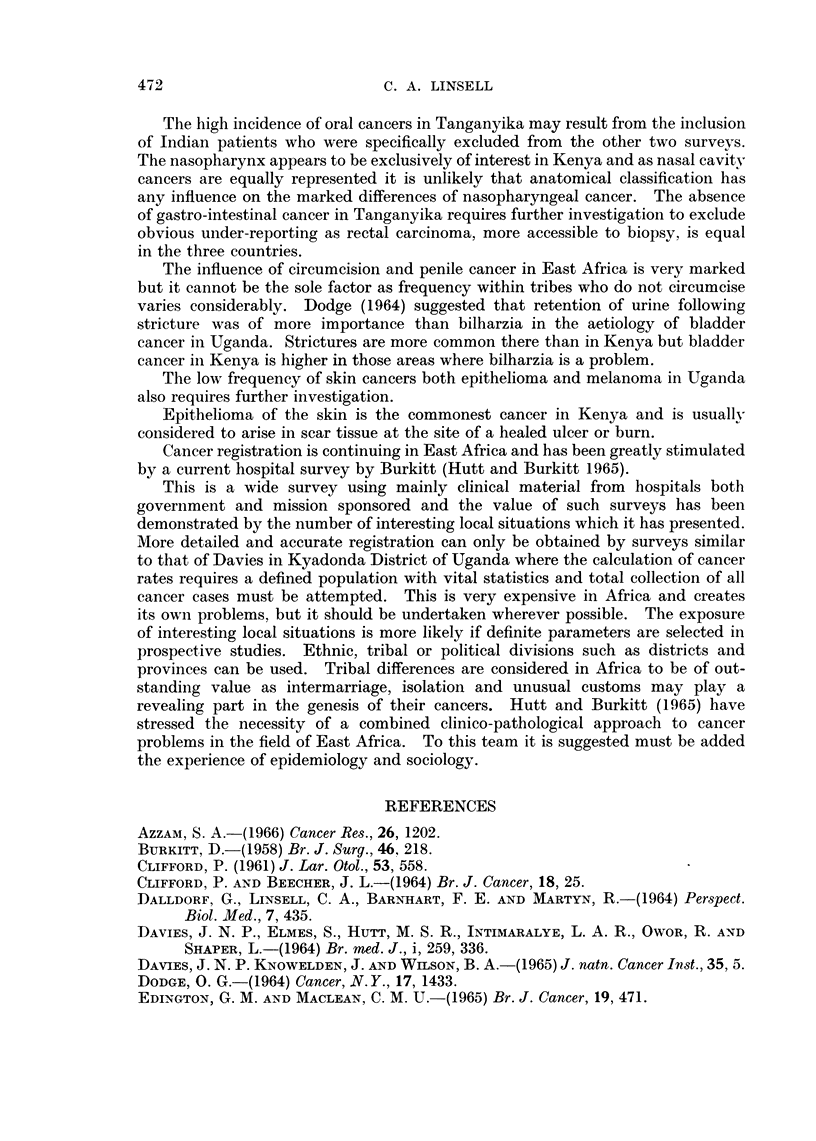

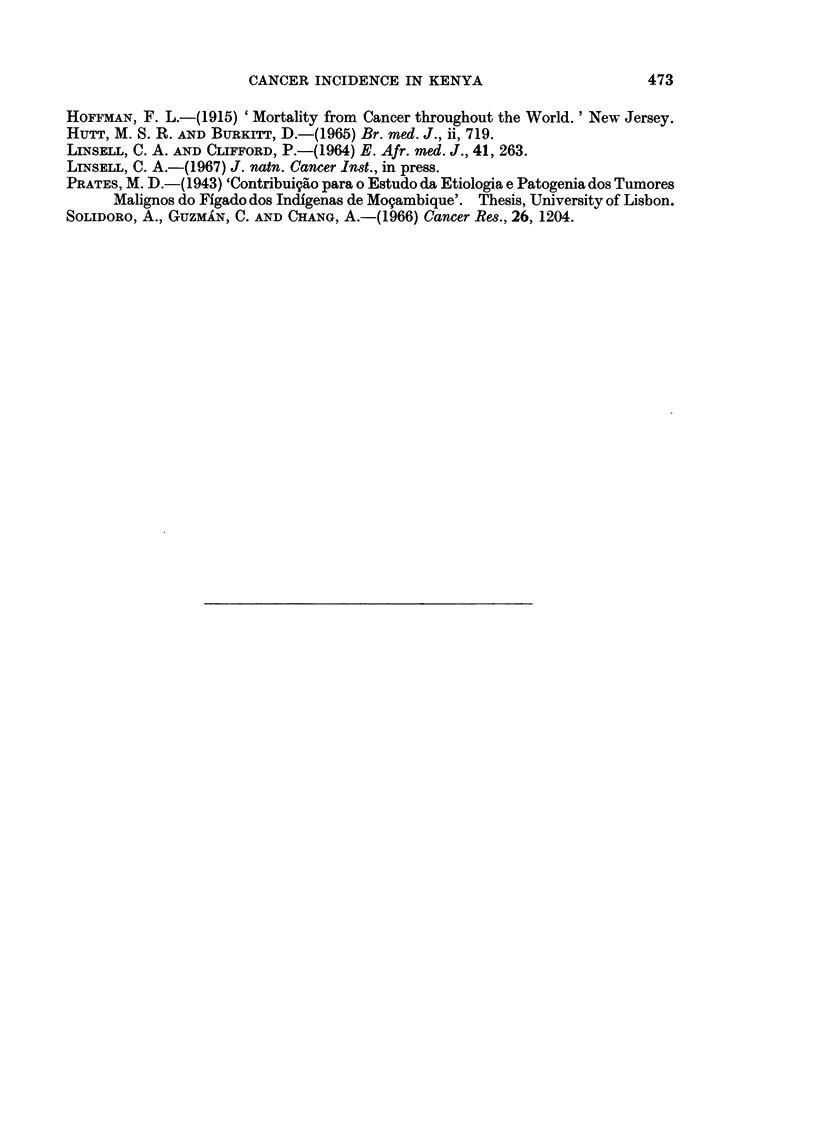


## References

[OCR_00918] Azzam S. A. (1966). High incidence of Hodgkin's disease in children in Lebanon.. Cancer Res.

[OCR_00919] BURKITT D. (1958). A sarcoma involving the jaws in African children.. Br J Surg.

[OCR_00926] DALLDORF G., LINSELL C. A., BARNHART F. E., MARTYN R. (1964). AN EPIDEMIOLOGIC APPROACH TO THE LYMPHOMAS OF AFRICAN CHILDREN AND BURKITT'S SACROMA OF THE JAWS.. Perspect Biol Med.

[OCR_00932] DAVIES J. N., ELMES S., HUTT M. S., MTIMAVALYE L. A., OWOR R., SHAPER L. (1964). CANCER IN AN AFRICAN COMMUNITY, 1897--1956. AN ANALYSIS OF THE RECORDS OF MENGO HOSPITAL, KAMPALA, UGANDA. 2.. Br Med J.

[OCR_00935] DODGE O. G. (1964). TUMORS OF THE BLADDER AND URETHRA ASSOCIATED WITH URINARY RETENTION IN UGANDA AFRICANS.. Cancer.

[OCR_00940] Hutt M. S., Burkitt D. (1965). Geographical distribution of cancer in East Africa: a new clinicopathological approach.. Br Med J.

[OCR_00942] LINSELL C. A., CLIFFORD P. (1964). TUMOURS OF THE LACRIMAL GLAND IN KENYA.. East Afr Med J.

[OCR_00948] Solidoro A., Guzmán C., Chang A. (1966). Relative increased incidence of childhood Hodgkin's disease in Peru.. Cancer Res.

